# A universal curve of apatite crystallinity for the assessment of bone integrity and preservation

**DOI:** 10.1038/s41598-018-30642-z

**Published:** 2018-08-13

**Authors:** Gregorio Dal Sasso, Yotam Asscher, Ivana Angelini, Luca Nodari, Gilberto Artioli

**Affiliations:** 1 Dipartimento di Geoscienze, Università degli Studi di Padova, Via G. Gradenigo 6, 35131 Padova, Italy; 2 Dipartimento dei Beni Culturali: archeologia, storia dell’arte, del cinema e della musica, Università degli Studi di Padova, Piazza Capitaniato 7, 35139 Padova, Italy; 3 Istituto di Chimica della Materia Condensata e di Tecnologie per l’Energia – ICMATE – Consiglio Nazionale delle Ricerche, Corso Stati Uniti 4, 35127 Padova, Italy

## Abstract

The reliable determination of bioapatite crystallinity is of great practical interest, as a proxy to the physico-chemical and microstructural properties, and ultimately, to the integrity of bone materials. Bioapatite crystallinity is used to diagnose pathologies in modern calcified tissues as well as to assess the preservation state of fossil bones. To date, infrared spectroscopy is one of the most applied techniques for bone characterisation and the derived infrared splitting factor (IRSF) has been widely used to practically assess bioapatite crystallinity. Here we thoroughly discuss and revise the use of the IRSF parameter and its meaning as a crystallinity indicator, based on extensive measurements of fresh and fossil bones, virtually covering the known range of crystallinity degree of bioapatite. A novel way to calculate and use the infrared peak width as a suitable measurement of true apatite crystallinity is proposed, and validated by combined measurement of the same samples through X-ray diffraction. The non-linear correlation between the infrared peak width and the derived ISRF is explained. As shown, the infrared peak width at 604 cm^−1^ can be effectively used to assess both the average crystallite size and structural carbonate content of bioapatite, thus establishing a universal calibration curve of practical use.

## Introduction

Bone is a composite material constituted by the intimate association of an organic matrix and a mineral phase arranged in a complex structure described in terms of hierarchical levels of organization^1,2^. Bioapatite, constituting the mineral fraction of bone, is a nano-crystalline phase, whose composition resembles that of hydroxylapatite (Ca_10_(PO_4_)_6_(OH)_2_)^3^ but considerably departs from the ideal end-member in terms of stoichiometry, as a number of ionic substitutions and vacancies occur in the structure^4–6^. The *in vivo* chemical composition and crystal structure of bioapatite are controlled by metabolic processes in order to fulfil specific functions^7,8^ as well as to regulate physical properties, such as solubility, thermal stability and crystal size^4,9^. The chemical and crystallographic characterization of bioapatite is of great interest for several research fields as archaeological science and palaeoclimatology^10–13^, as well as medicine and biomedical engineering^6,14–18^. Among the measurable properties of bioapatite, crystallinity is an extremely meaningful indicator of bone preservation^11,13^ in the case of fossil bones and of integrity^15,19–21^ in the case of fresh bones. In fact, nanocrystals are thermodynamically unstable and are prone to recrystallization both during the lifetime of the individual and after death. After the death of the individual bioapatite crystals tend to recrystallize by Ostwald ripening process into a phase characterized by a more stable and more ordered crystal structure and by larger crystal dimensions, implying a reduced specific surface area^11,22,23^. Variability in the recrystallization degree is determined by the combined effects of several factors, mainly related to environmental and local burial conditions, such as soil composition, pore water chemical composition, temperature and moisture^11,24,25^. Therefore, the physico-chemical characterisation of fossil bones is crucial for those studies aiming to assess their pristine composition and diagenetic history as well as to reconstruct past climate conditions^23,25–30^. However, the term crystallinity is loosely used to describe the properties of the material related to crystal structure order, density of defects, or crystal size and shape. In order to describe or quantify the changes in bioapatite crystallinity, several empirical crystallinity parameters have been proposed, on the basis of results obtained from different analytical techniques such as X-ray powder diffraction – XRPD –^31–34^, Fourier transform infrared spectroscopy – FTIR –^35,36^, Raman spectroscopy^15,37,38^, transmission electron microscopy – TEM –^28,39^, atomic force microscopy^40,41^, nuclear magnetic resonance – NMR –^40^. Among these techniques, FTIR spectroscopy is extensively applied to bone characterization studies^6,13,42^ as it provides valuable information on the chemical composition and structure of both the mineral and organic phases, it requires a low amount of sample (~1 mg) and the analytical procedure is fast and nearly inexpensive, so that a large set of samples can be feasibly investigated. Hence, the infrared splitting factor (IRSF) calculated from FTIR spectra of bone samples^10,35^ is a widely used parameter to practically assess crystallinity. The IRSF is a function of infrared peak broadening and it quantifies the splitting extent of the two main peaks of the phosphate ν_4_ vibrational mode. Peak broadening is, in turn, inversely related to crystallinity as a more crystalline apatite is characterised by sharper infrared peaks, and thus by a higher IRSF value^10,35,43^.

Given the relevance gained by this diagenetic parameter to assess the preservation state of fossil bones, in this research a detailed study on the relationship between IR peak width, IRSF and crystallinity has been carried out by investigating fresh and archaeological bones. This set of samples covers a wide range of crystallinity as fresh bones are inherently characterized by poorly crystalline bioapatite, whereas archaeological bones from different sites show a large variability in terms of recrystallization degree, depending on the types and extent of alteration processes encountered during burial. The effectiveness of IRSF as a parameter for describing crystallinity variations is here discussed by comparing results obtained from FTIR spectroscopy with an independent measurement of bioapatite crystallinity carried out by X-ray powder diffraction coupled with the Rietveld refinement of diffraction data, being this technique the most suitable and statistically robust to assess the average size of bioapatite crystallites. On these bases, the ultimate aim of this research is to provide a tool to reliably assess bioapatite crystallinity variations among a set of samples, encouraging the use of analytical techniques of easy accessibility, such as FTIR spectroscopy.

## Remarks on Bioapatite Crystallinity

Due to the complex nature of biogenic apatite, a unique appropriate method for crystallinity determination is not established; conversely, several indicators, providing some relative measurement of crystallinity, are commonly used and derived from a number of analytical techniques and from different approaches to data analysis^6,10,15,28,33,34,36,37,39,40^. Different analytical techniques probe different physical-chemical properties of the analysed material, thus these indicators can be directly or indirectly related to crystallinity. This results in an unclear physical meaning of some of these indicators, often related to a loose definition of crystallinity. In this research bioapatite crystallinity is investigated by FTIR spectroscopy, and, for a subset of samples, by XRPD. Since these two techniques rely on different interaction processes between radiation and the analysed material, crystallinity is assessed trough a substantially different approach. For this reason, defining the term *crystallinity* when referring to biogenic apatite and the physical meaning of crystallinity indicators is fundamental^44^.

The most basic definition of crystal refers to a solid material “having an essentially discrete diffraction diagram”^45^ (see also the Online Dictionary of Crystallography at http://reference.iucr.org/dictionary/Crystal); it includes a vast class of solid materials characterized by a long-range regular and periodic arrangement of atoms. Generally speaking, the term *crystallinity* refers to the degree of structural order in a solid material; such order can be moderated by the occurrence of crystal defects and grain boundaries. Depending on the analysed material and the specific application required, the term crystallinity may assume different meanings, i.e. the amount of crystal defects in a single crystal, the crystallites dimension in a polycrystalline material, or the volume percentage of crystalline and amorphous materials in a mixture. In the case of bone material the term crystallinity refers to the mean dimension of bioapatite nanocrystals. In fact, bioapatite can be considered a polycrystalline material, although it is often described as a poorly crystalline apatite because of the high specific surface area of crystals and the large amount of ionic substitutions occurring in its structure. Bioapatite crystallinity can be indicative for bone quality or preservation, since nanocrystals tend to recrystallize into larger crystals with a more ordered structure^11,13^ as soon as specific dysfunctions or the death of the individual hinder the metabolic processes normally controlling bioapatite chemical composition and crystal size^7,8,11^.

On these bases, bioapatite crystallinity is differently probed by FTIR spectroscopy and X-ray powder diffraction. Vibrational spectroscopies (i.e. FTIR and Raman spectroscopy) are sensitive to functional groups and their chemical environment, probing the vibrational frequencies associated to chemical bonds through the absorption and/or scattering of photons. The commonly employed IRSF is directly related to the broadening of the IR phosphate peaks referring to the ν_4_ vibrational mode. Broadening of these peaks is caused by length changes and distortions of the P-O bonds within the bioapatite structure due to the increase of crystal defects as substitutions, vacancies and strained lattice sites^9^. Two major effects are present in the case of bioapatite: (i) the high specific surface area due to the nanometre size of crystals, that contributes to change the average P-O bond length because of surface relaxation, and (ii) a significant contribution to peaks broadening resulting from the amount of carbonate substituting for phosphate, which is the most significant (4–7 wt.%) among the ionic substitutions occurring in the bioapatite crystal structure^4,9,46^. Furthermore, the significant occurrence of carbonate ions in the bioapatite structure implies a higher degree of structural disorder as several ions substitutions and vacancies are favoured to maintain the charge balance^6,47^. As proved by De Mul and co-authors^46^ in a detailed study, the peak width of the phosphate symmetric stretching vibration at 962 cm^−1^ in Raman spectra depends on the amount of carbonate substitution in the hydroxylapatite structure. Evidence of the dependence of phosphate peak width on the carbonate content is also provided by several studies showing the strong correlation between IRSF values and carbonate content retrieved by IR spectra of bones^25,36,37,48,49^. Even though peak broadening, and therefore the IRSF, is directly related to the carbonate content of bioapatite, the amount of ionic substitution and crystallite size are not independent of each other. In fact, the occurrence of carbonate ions at high concentration in the bioapatite structure influences the long-range atomic order of crystals through accumulated strain, thus inhibiting the crystal growth and constraining the bioapatite crystallites at a nanometre scale^4,9^. The correlation observed between the IRSF and the average crystallite size, measured on bone material by XRPD^50,51^ indicates that the IRSF provides a relative measurement of bioapatite crystallinity defined as mean crystallite size. Therefore, FTIR spectroscopy can be reliably applied to monitor bioapatite crystallinity, even if indirectly, with the advantages provided by this technique with respect to others, such as the small sample size required and the fast and nearly inexpensive analytical procedure. Within this framework, it is worth mentioning one of the first applications of the splitting quantification of the two phosphate peaks to monitor apatite crystallinity and frequently cited when analysing bone material through FTIR spectroscopy: Termine and Posner^43^ successfully developed a method to quantify crystallinity in apatitic calcium phosphates; however the term crystallinity here used refers to the ratio of crystalline apatite and amorphous calcium phosphate in a mixture, which is substantially different by the meaning of crystallinity referred to bioapatite previously discussed. In the cited case, the increase of peak width observed for amorphous phosphate with respect to that observed for crystalline apatite can be related to the larger distribution of the P-O bond lengths in the amorphous material with respect to that of well-ordered crystalline material.

On the other hand, X-ray powder diffraction (XRPD) directly probes bioapatite crystallinity as the crystallites act as coherently scattering domains producing a diffraction pattern. The nanometre size of crystallites causes the broadening of line profiles^52–54^, thus by line-broadening analysis the average crystallite dimensions along different crystallographic directions can be calculated. Depending on the approach used to analyse diffraction data, crystallinity can be monitored by taking into account the whole diffractogram^34,50,51^ or through other crystallinity indicators based on single peak width measurement^31^ or averaged among several crystallographic directions, the meaning of which, however, may result unclear^33^. The more rigorous whole profile fitting method has the advantage to provide a more accurate determination of apparent crystallites dimensions along several crystallographic directions, thus taking into account the intrinsic anisotropy of bioapatite crystallites. In this research, the Rietveld refinement method^55^ was applied to diffraction data in order to retrieve the average apparent crystallites dimensions, considering the anisotropic contribution to the line broadening due to crystallite size. Since the direction of maximum elongation is along the c axis, the crystallite size along this direction is here considered as a proxy for crystallinity and used as a parameter to monitor crystallinity variations among samples.

## FTIR Spectral Analysis

Bioapatite samples were selected in order to cover a wide range of crystallinity, in particular they consist of fresh bone and dentine samples, providing a reference for bioapatite produced by metabolic processes in mammals^13^, and archaeological bones characterised by variable recrystallization degrees selected from different sites located in Israel and in Sudan.

**Figure 1:**
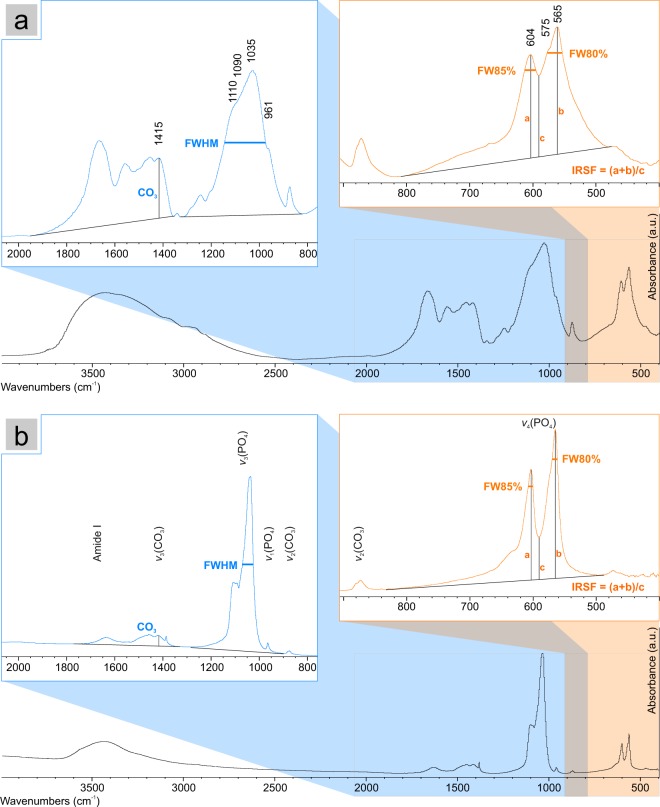
FTIR spectra of (**a**) fresh bone and (**b**) diagenetically altered archaeological bone. Baselines, the full width at half maximum (FWHM) of the phosphate peak at 1035 cm^−1^, the width at 85% of height of the peak at 604 cm^−1^ (FW85%), the width at 80% of height of the peak at 565 cm^−1^ (FW80%) and the splitting factor (IRSF) are highlighted. Major vibrational bands attribution is also reported (see the Supplementary Information for a detailed description).

FTIR spectroscopy was carried out on the entire set of samples. The raw FTIR spectra (Supplementary Fig. S1) and the detailed interpretation of vibrational modes detected in bone FTIR spectra are reported in the Supplementary Information online. Several parameters, i.e. peaks intensity ratio and width, were calculated from each FTIR spectrum to monitor the peak shape and relative intensity variations among samples. Spectra manipulations, as smoothing, background subtraction or peak deconvolution, were avoided; nevertheless, all calculated parameters (peaks intensity and width) refer to a baseline (as shown in Fig. 1) in order to make them comparable^25,36^. The baseline (Fig. 1) was defined for each spectrum by a number of points placed at the local minimum within selected regions of the spectrum, as reported in Table 1.

**Table 1:** Peak positions and baselines of vibrational bands used to calculate peaks intensity and width.

Vibrational mode	Maximum peak position (cm^−1^)	Baseline (cm^−1^)
ν_3_(CO_3_)	1415	2000–1800/1400–1200
ν_3_(PO_4_)	1035	1400-1200/900-750
ν_4_(PO_4_)	604	850-620/510-470
ν_4_(PO_4_)	565	850-620/510-470

For each peak the baseline is defined by two points selected at the local minimum in the spectral region within the intervals here indicated (Fig. 1).

Even though spectra deconvolution is a preferable technique to isolate each single contribution of overlapping bands^56^, in this study a different approach is suggested and based on the selection of specific features less affected by overlapping effects. This choice is driven by two reasons: (i) the large success of IRSF as a practical parameter for bioapatite crystallinity lies on the easy way to calculate it, avoiding spectra deconvolution that is a complex and time-consuming (even though reliable) method; (ii) the selection of suitable parameters, measured directly from FTIR spectra, can provide results that are as reliable as those obtained from spectra deconvolution (The close comparison between spectral deconvolution and measurable spectral parameters is reported in the Supplementary Information, Supplementary Fig. S2). Since this research aims to provide a reliable but easily applicable method to monitor crystallinity variations among samples, a set of parameters was calculated from FTIR spectra as described in the following paragraph.

The **IRSF**^35^ is calculated as the sum of peak intensities at 604 and 565 cm^−1^ (ν_4_(PO_4_) vibrational mode) divided by the intensity of the valley between them (i.e. (a + b)/c as reported in Fig. 1); phosphate peaks were also characterised by measuring the full width at half maximum (**FWHM**) of the main peak at 1035 cm^−1^ referring to the ν_3_(PO_4_) vibrational mode, the width at 85% of the height of the 604 cm^−1^ peak (**FW85%**) and at 80% of the height of the 565 cm^−1^ peak (**FW80%**). The peaks height here considered is calculated from the baseline previously defined (Fig. 1). The height percentage used to calculate FW85% and FW80% was selected considering the bands overlapping among all the samples, thus 85% and 80% was the minimum percentage allowing the measurement of the width of the sole 604 cm^−1^ and 565 cm^−1^ peak, respectively.

The carbonate content of bioapatite is monitored through the **CO**_**3**_**/PO**_**4**_ parameter, calculated by dividing the intensity of the band at 1415 cm^−1^ (ν_3_(CO_3_) vibrational mode) by the intensity of the phosphate peak at 604 cm^−1^ (i.e. CO_3_/a, as shown in Fig. 1). Even though carbonate ions in nano-apatite occur in three different structural environments (named A-type, when substituting for OH^−^ ions, B-type, when substituting for PO_4_^3−^ ions, and labile carbonate – see Supplementary Information for a detailed description), the B-type carbonate is the predominant one in bone mineral, whereas the A-type is much less abundant and the labile one is occasionally detected^4,9,57^; moreover the ratio between A- and B-type carbonate is nearly constant^57^. Therefore, the semi-quantitative estimation of carbonate content based on FTIR parameters that consider only the vibrational bands referring to the B-type carbonate (as the CO_3_/PO_4_ parameter based on the band at 1415 cm^−1^) can be considered to be proportional to the total structural carbonate content of bioapatite. This is furthermore proved by other studies^58–60^ showing that the total amount of carbonate, quantified analytically, is linearly well-correlated to the CO_3_/PO_4_ parameter previously described. Therefore, the CO_3_/PO_4_ parameter can be effectively applied to estimate the relative amount of carbonate content in bioapatite samples.

## Results

**Figure 2:**
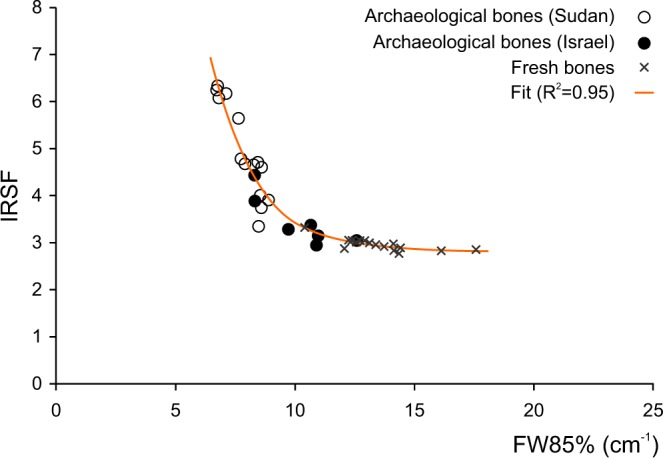
Correlation between the IRSF and FW85% among bioapatite samples and plot of the regression function.

Results (Supplementary Table S1) show a high variation of FW85% values for the less crystalline bioapatite (fresh bone and dentine), ranging from 10.4 to 17.6 cm^−1^; conversely, the variation of IRSF values, ranging from 2.8 to 3.3, is minimal. Archaeological bones from Israel and Sudan show higher variations of IRSF with respect to FW85%: IRSF ranges from 2.9 to 4.4 and from 3.3 to 6.3, respectively, whereas the FW85% ranges from 8.3 to 12.6 cm^−1^ and from 6.7 to 8.9 cm^−1^, respectively. Archaeological bones from Sudan show significantly higher IRSF and lower FW85% than those of fresh bones; within this range, data referring to archaeological bones from Israel partially overlap to those of fresh bones and of archaeological bones from Sudan (Fig. 2). FW80% ranges from 19.2 to 32.0 cm^−1^, from 14.5 to 22.3 cm^−1^ and from 8.2 to 19.1 cm^−1^, whereas FWHM ranges from 110.4 to 171.4 cm^−1^, from 89.8 to 120.9 cm^−1^ and from 43.5 to 94.7 cm^−1^ for fresh bones, archaeological bones from Israel and Sudan, respectively. The CO_3_/PO_4_ parameter was calculated for all samples with the exception of archaeological ones for which the occurrence of secondary calcite hampers a reliable determination of the structural carbonate content of bioapatite. In fact, the carbonate vibrational modes of the bioapatite structural carbonate are superimposed on those of calcium carbonate^25^. The CO_3_/PO_4_ ranges from 0.5 to 0.9 and from 0.3 to 0.5 for fresh and archaeological bones, respectively.

Repeated measurements on the same sample provided a relative standard deviation below 5% for these parameters.

**Figure 3:**
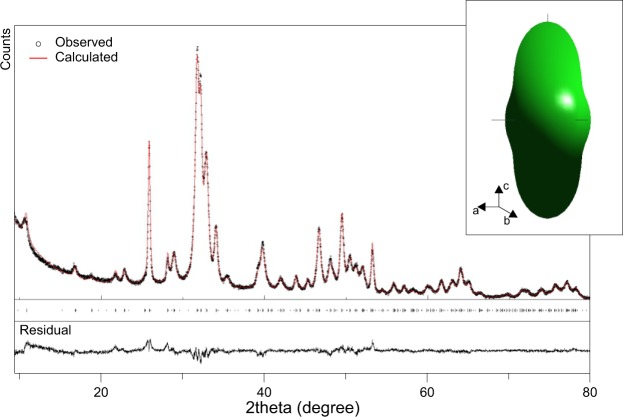
Diffractogram of an archaeological bone sample from Sudan. The calculated pattern and the residual resulting from the Rietveld refinement (Rwp = 4.45%) is also displayed. The top-right panel shows the average shape of bioapatite crystallites, elongated along the c-axis, reconstructed from the spherical harmonics coefficients refined by Rietveld method.

A subset of samples was also analysed by XRPD and diffractograms were analysed by Rietveld refinement (Fig. 3). The quality of Rietveld refinement was monitored by the residuals and by the agreement factor (Rwp), at 6% for fresh bones and lower than 5% for archaeological bones. The crystallite size along the c axis was determined, ranging from 21 to 25 nm and from 37 to 71 nm for fresh bones and archaeological bones from Sudan, respectively (Supplementary Table S1). The crystallite size along the a axis is also reported, ranging from 9 to 14 nm and from 15 to 32 nm for fresh and archaeological bones, respectively. As a general trend, crystallites sizes increase both along the c and a axes. The relative standard deviation associated to crystallite sizes is within 3%. The anisotropic contribution to line broadening due to microstrain appears to be negligible along the c axis, whereas a more significant contribution was determined along the a axis (Supplementary Table S1).

## Discussion

The set of bioapatite samples here analysed shows a high variability in terms of chemical and structural properties, that is due both to the expected variability occurring in fresh bones (different types of bone/different taxa) and to the different recrystallization pathways affecting archaeological bones during diagenesis. FTIR spectra reflect this variability, as several features such as peaks width, shape and position significantly change within this set of samples. A fresh bone and a heavily altered archaeological bone from Sudan are chosen here as an example to show the extent of such variability (Fig. 1). The previously described parameters FWHM, FW85% and IRSF are graphically reported in Fig. 1b.

**Figure 4:**
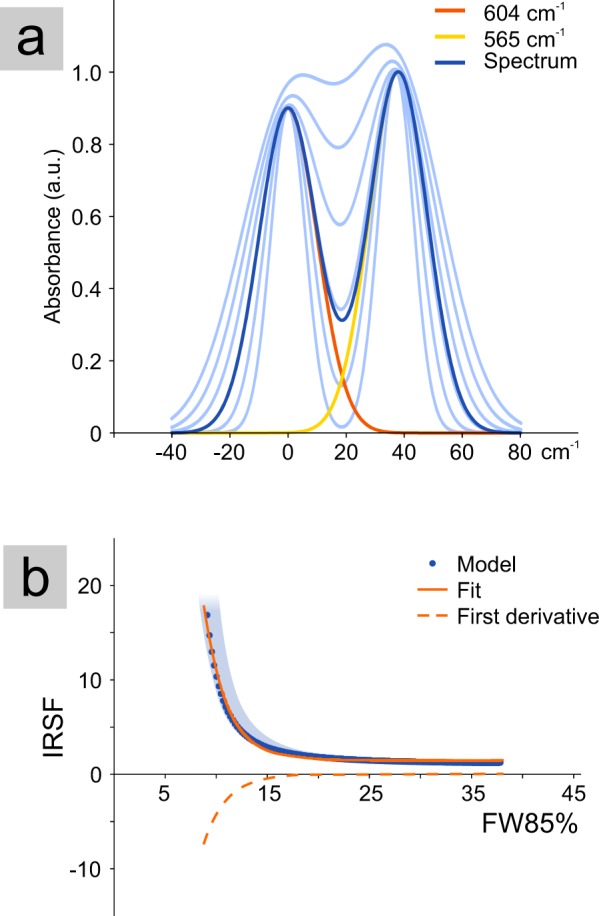
(**a**) Simplified model of the ν_4_(PO_4_) vibrational mode representing the IR spectra (blue) resulting from the contribution of the 604 and 565 cm^−1^ peaks, approximated by Gaussian curves; in light-blue is represented the change of the spectrum shape by varying the width of the two Gaussian curves. (**b**) Plot of the calculated IRSF against the width at 85% of the height of the modelled peaks; an exponential regression function fits the calculated points (R^2^ = 0.996) and its first derivative shows the curve slope; the light blue area shows the variation range of IRSF and FW85% considering a possible shift of the two peaks centred position, resulting in a variation of the measured distance between the two maxima in the range 37–43 cm^−1^ (according to the experimental dataset).

The plot of the IRSF against the phosphate peak width (FW85%) is a useful tool to monitor the variation of these two parameters among the entire set of samples (Fig. 2). The IRSF is a widely used parameter to describe crystallinity variations, whereas the use of the width of the phosphate peak at 604 cm^−1^ is scarcely attested^14,42^. Considering a limited set of samples, as fresh bone samples or bones coming from a single archaeological site could be, a substantially good linear correlation can be observed between IRSF and FW85%. However, when considering a larger set of samples, that covers a wide range of variability in terms of crystallite size and chemical composition, a non-linear correlation between these two parameters is clearly observable (Fig. 2). The decrease of the phosphate peak width (FW85%) is followed by a non-linear increase of IRSF. This behaviour is artificial and it is due to the method used to calculate the IRSF rather than to a significant change in some bioapatite properties other than the sharpening of peaks. This is clearly proved by a numerical simulation of the ν_4_(PO_4_) vibrational mode (Fig. 4a) showing the variation of the IRSF with peak width (Fig. 4b).

Here a simplified model of the bioapatite spectrum is calculated as the sum of the main phosphate peaks at 604 and 565 cm^−1^ (referring to the positions at 0 and 39 cm^−1^, respectively), the relative intensity of which is normalised to 1, and described by a Gaussian function in the form:

1 $$y=a\,ex{p}^{\frac{-{(x-b)}^{2}}{2{c}^{2}}}$$

where *a* is the height of the Gaussian peak, *b* is the position of the peak centre and *c* is related to the width of the peak. The width of the two peaks is equal and the width at 85% of the height is calculated as:

2 $$FW85 \% =2\sqrt{ln{0.85}^{-2}}c$$

The IRSF^35^ is calculated as the sum of the two peaks heights divided by the height of the valley between them. By decreasing the value of the variable c, the peaks progressively sharpen, thus FW85% decreases and IRSF increases. The relationship between the width of the two peaks and the related IRSF (Fig. 4b) can be reasonably described by a regression model (R^2^ = 0.99) with an exponential function of the type:

3 $$IRSF=p\,ex{p}^{q[FW85 \% ]}+r$$

where *p*, *q* and *r* are real constants. The first derivative of this equation is itself an exponential function and describes the changing slope of the FW85%-IRSF correlation with peak sharpening. This model clearly shows that the IRSF is non-linearly correlated with the peaks width used to calculate it.

Back to the experimental data, the relationship between the experimental FW85% and IRSF similarly behaves to that observed in the numerical model. The regression function (R^2^ = 0.95) of the experimental data, obtained by least squares method according to the Equation (3), shows that the numerical model, even if simplified, describes the correlation between the two parameters (Fig. 2). The different behaviour of fresh bone and dentin with respect to that of archaeological bone in terms of correlation between peak width and IRSF is completely artificial and depends on the IRSF calculation method. It is now clear that a constant sharpening rate of the phosphate peaks correspond to an exponential increase of the IRSF. This highlights that the IRSF shows a different sensitivity in describing the variations of phosphate peaks width: IRSF does not show any significant variation for most of the fresh bone and dentin samples with respect to the peak width sharpening, conversely for archaeological bones slight variations of peak width correspond to high IRSF variation. Since the source of physical information regarding the bioapatite structure is the width of the IR phosphate peaks, the choice of the IRSF to monitor bioapatite crystallinity, even if widely used, may hamper a straightforward and reliable comparison of different samples.

**Figure 5:**
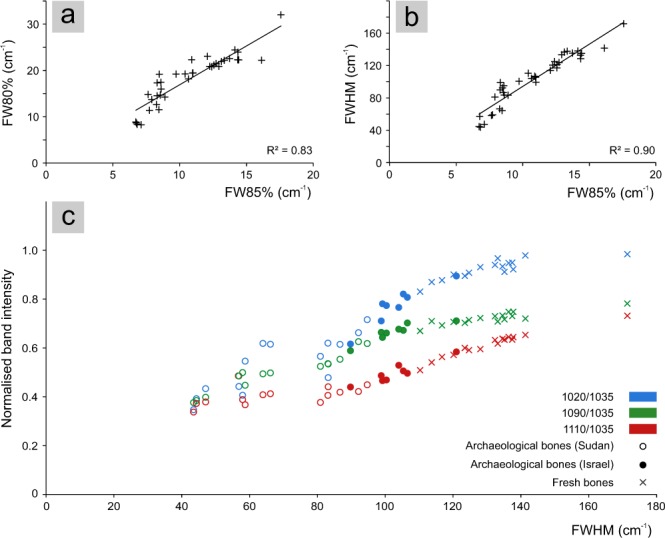
Plot showing the correlation between (**a**) the width at 80% of the height of the 565 cm^−1^ peak (FW80%) and the width at 85% of the height of the 604 cm^−1^ peak (FW85%); (**b**) the FWHM of the 1035 cm^−1^ peak and the width at 85% of the height of the 604 cm^−1^ peak (FW85%); (**c**) the variation of the 1020, 1090 and 1110 cm^−1^ bands normalised to the 1035 cm^−1^ band among all bioapatite samples. The relative standard deviation for these parameters is within 5%.

It is worth reminding that all phosphate peaks are actually a reliable source of information on bioapatite structure; this is consistent with the linear correlation observed between the width of the main phosphate peaks at 1035, 604 and 565 cm^−1^ (Fig. 5a,b), monitored through the FWHM, FW85% and the FW80%, respectively. The choice of using the width of the 604 cm^−1^ peak at 85% of the height (FW85%) was driven by the complexity of the phosphate vibrational modes and therefore by the number of vibrational bands contributing to the width measurement of the phosphate peaks. Within the ν_4_(PO_4_) vibrational mode, mainly characterized by three vibrational bands at 604, 575 and 565 cm^−1^, the one at 604 cm^−1^ produces a well-defined peak; by measuring its width at 85% of the height the contribution from other overlapping bands is eliminated, thus the peak width (FW85%) is due to a single vibrational band, a unique case for bioapatite IR spectra. Conversely, the width of the 565 cm^−1^ peak measured at 80% of the height (FW80%) is influenced by the 575 cm^−1^ band, occurring as a shoulder, the relative intensity of which significantly varies among samples with respect to the 565 cm^−1^ band, from 0.7 to 0.97. Therefore, in some cases it contributes to the FW80% measurement, whereas it does not contribute to the width measurement when the intensity ratio is below 0.8. This partially affects the linearity of the correlation between FW85% and FW80%, as observed in Fig. 5a. Similarly, FWHM measures the width of the main phosphate peak at 1035 cm^−1^, which results from the contribution of several vibrational bands constituting the ν_3_(PO_4_) vibrational mode; these vibrational bands, associated to different environments or configurations of phosphate ions that coexist in the bioapatite structure^61^, differently contribute to the peak shape depending on the stoichiometry and structure of the analysed bioapatite. This accounts for a poorer linear correlation with FW85% (Fig. 5b). In order to simplify the discussion of data only few vibrational bands are here considered, in particular the 1090 and 1035 cm^−1^ bands associated to stoichiometric hydroxyapatite and the 1110 and 1020 cm^−1^ bands referring to phosphate ions in a non-stoichiometric apatite^61^. The gap observed in the range 65–80 cm^−1^ of FWHM (Fig. 5b,c) is mainly associated to the significant change of the peak shape due to the different contribution of the band at 1090 cm^−1^ when analysing less ordered and more ordered bioapatite. For less ordered bioapatite, the peak at 1090 cm^−1^ appears as a shoulder and it contributes to the width measurement at half maximum of the main peak at 1035 cm^−1^ (FWHM), whereas for more ordered bioapatite these two bands are clearly separated in two peaks and the 1090 cm^−1^ band does not contribute to the FWHM measurement (Fig. 1). In fact, the ratio between the 1090 cm^−1^ and the 1035 cm^−1^ bands among samples varies from 0.38 to 0.80. Therefore, the gap between 65 and 80 cm^−1^ occurs at the transition point in which the 1090 cm^−1^ band is included in the FWHM measurement (values higher than 80 cm^−1^) or excluded (values lower than 65 cm^−1^). The plot of the 1020, 1090 and 1110 cm^−1^ bands normalised to the 1035 cm^−1^ band against the FWHM of the 1035 cm^−1^ peak (Fig. 5c) shows the different variation of vibrational bands contribution with respect to the width of the peak as well as with respect to different types of bioapatite. This shows that the sole width of the main phosphate peak cannot be reliably used to monitor chemical/structural variations among samples because of the complexity of vibrational bands contributing to the ν_3_(PO_4_) vibrational mode.

**Figure 6:**
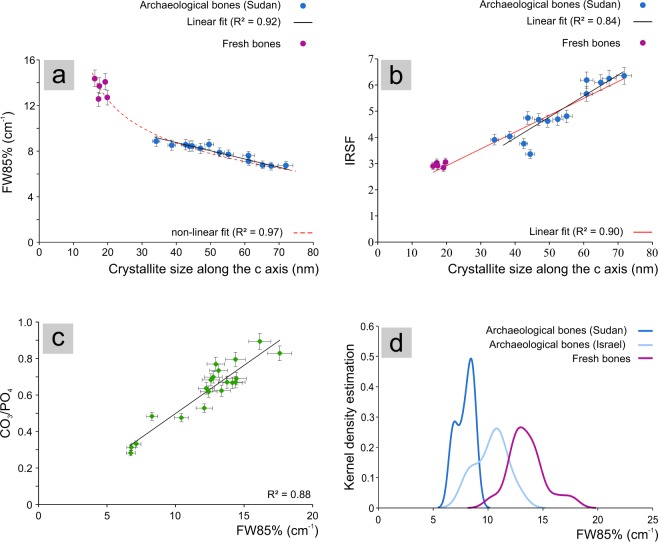
Plot showing (**a**) the non-linear correlation between the FW85% (FTIR spectroscopy) and the crystallite size along the c axis (XRPD); a tentative exponential fit is also displayed. The approximated linear correlation between the two parameters for archaeological bones from Sudan (R^2^ = 0.92) is reported; (**b**) the linear correlation between IRSF (FTIR spectroscopy) and the crystallite size along the c axis (XRPD) considering the entire set of samples (R^2^ = 0.90) and a subset of samples constituted by archaeological bones from Sudan (R^2^ = 0.84); (**c**) Plot showing the correlation between the FW85% and the structural carbonate content of bioapatite; CO_3_/PO_4_ parameter was calculated for fresh bones and for a subset of archaeological bones not contaminated by secondary calcite. (**d**) Kernel density estimation of the FW85% parameter measured for all samples. Error bars show the relative standard deviation estimated for FTIR parameters (5%) and crystallite size (3%).

On these bases, the width of the 604 cm^−1^ peak at 85% of the height (FW85%), being less affected by peaks overlapping effects, is the most suitable parameter to describe the variation of bioapatite structural properties within a set of samples. The comparison of results obtained from FTIR spectroscopy and XRPD enables one to verify the correlation between the FW85% parameter and the actual bioapatite crystallite size. It is worth to mention that IR phosphate peak width provides vibrational information that is averaged along the crystallographic directions and does not take into account the anisotropic nature of the bioapatite crystals. Conversely, the Rietveld refinement performed on diffraction data provides the crystallite dimensions along several crystallographic directions taking into account the anisotropic contribution to the line broadening due to crystallite size, therefore describing crystallinity as an anisotropic property of bioapatite. Nevertheless, considering that the direction of maximum elongation of bioapatite crystallites is along the c axis and that the (*00 l*) reflections in the diffraction pattern are not overlapped by other peaks (thus being easily modelled and providing more reliable results), the crystallite size (expressed in nm) retrieved along the c axis was selected as a single and suitable parameter for crystallinity to be compared with FW85%. Therefore, a subset of samples covering the entire range of FW85%-IRSF (fresh bones and archaeological bones from Sudan) was analysed by XRPD and for each sample the crystallite dimension along the c axis was compared to the FW85% parameter (Fig. 6a). Results show a non-linear correlation between FW85% and crystallite size, as the less crystalline fresh bones show a faster increase of FW85% with the decrease of crystallite size with respect to archaeological bones (Fig. 6a). Conversely, the IRSF and the crystallite size are linearly correlated through the entire set of samples (Fig. 6b). This different behaviour is consistent with the non-linear correlation between FW85% and IRSF previously discussed. In fact, FW85% and crystallite size could be tentatively correlated through an exponential function (Fig. 6a). When considering a subset of samples with a reduced crystallinity variation, as the case of archaeological bones from Sudan characterized by a high recrystallization degree, the non-linear correlation between FW85% and crystallite size can be reliably approximated (R^2^ = 0.92) to a linear one (Fig. 6a). In this case, also the IRSF and the crystallite size are linearly correlated (Fig. 6b), but the quality of the linear fit is significantly lower (R^2^ = 0.84). The large dispersion of data with respect to the regression model shows that the IRSF cannot accurately monitor small variations of crystallite size, as the FW85% parameter do, if a subset of samples with a reduced crystallinity variation is considered.

Noteworthy, the width of phosphate peak FW85% is linearly correlated to the amount of structural carbonate in the bioapatite lattice (Fig. 6c), the relative measurement of which is provided by the CO_3_/PO_4_ parameter. As previously discussed, this furthermore proves that the width of the phosphate peak significantly depends on the carbonate content of bioapatite. It is thus not surprising that a clear correlation is detected between the phosphate peak width and the crystallite size, since the occurrence of structural carbonate plays a major role in controlling the size of crystallites in bioapatite^9^. Moreover, it is largely attested in the literature the loss of structural carbonate for archaeological bones after recrystallization processes^11^.

These results show that both the FW85% and IRSF can be effectively used to monitor significant variations of bioapatite crystallinity among samples; however, the FW85% parameter is more accurate in describing smaller variations. The width of the 604 cm^−1^ peak is an effective parameter to differentiate bioapatite samples characterised by different chemical and structural properties. The statistical analysis of FW85% values through the Kernel Density Estimation (KDE) may be an effective way to describe the distribution of this parameter measured for the three groups of samples here analysed (fresh bones and archaeological bones from Israel and Sudan). Using the software developed by the Analytical Methods Committee of the Royal Society of Chemistry (www.rsc.org/amc/), each value for FW85% is replaced by a normal function centred on the point and with a standard deviation depending on the smoothing parameter (h), that is statistically determined^62^. The the sum of the normal distributions scaled to have a unit area is the KDE. This function provides an estimate of the FW85% density function for each group of samples without any assumption on its distribution. The KDE of FW85% values (Fig. 6d) shows significant variations among the three sample groups in terms of frequency distribution of the phosphate peak width. This is extremely meaningful as fresh bones can be easily distinguished from the altered ones on the basis of the phosphate peak width, and, even within the archaeological bone samples, differences in terms of alteration/recrystallization degrees can be identified. Archaeological bones from Sudan and from Israel, that underwent significant different diagenetic processes, show different ranges of variation in terms of phosphate peak width; moreover, this parameter can further distinguish between different extents of the recrystallization processes within a set of samples from the same site. In the case of Sudanese bones for example, the bimodal frequency distribution of the phosphate peak width (Fig. 6d) is related to bones of different burial phases that experienced different diagenetic processes due to the change of climatic and burial conditions^25^.

These results suggest that the IRSF is not the most recommended parameter for monitoring bioapatite crystallinity, even if it has been a well-established and used parameter. The IRSF can still roughly provide a description of crystallinity variations when comparing different samples but it is far less accurate than the width of the phosphate peak at 604 cm^−1^. The width of the 604 cm^−1^ peak shows a linear correlation with the structural carbonate content of bioapatite and a non-linear correlation with the crystallite size. Therefore, the measurement of the phosphate peak width, once established a proper calibration curve based on these correlations, can be associated to both the chemical composition and crystallite size of bioapatite, thus providing a reliable assessment of these important material properties.

## Conclusions

The use of a large set of bioapatite samples characterised by a wide range of crystallinity, from fresh samples to extremely recrystallized fossil bones, allowed to clarify the nature and physical meaning of the empirical parameters frequently used to evaluate the preservation state of bone materials. The main aim of this study is to provide a reliable method to retrieve significant physical information on material properties, such as crystallinity, that are indirectly investigated with analytical techniques of easy accessibility, in this case FTIR spectroscopy. This research describes for the first time the exponential nature of the correlation of the IR phosphate peaks width with the splitting factor (IRSF) and the non-linear correlation with the mean crystallite size of bioapatite. The results show the limitations of the IRSF to describe the variation of peaks width and to reliably compare samples with different crystallinity. Conversely, this study highlights the relevance of phosphate peaks width for monitoring minimal changes in bioapatite chemical composition and structure. It is shown that coupling FTIR measurements with other quantitative analyses, thus establishing appropriate calibration curves, the width of the peak at 604 cm^−1^ can provide reliable values of mean crystallite size and structural carbonate content for biogenic apatite. On a wider perspective, this method can be practically applied to monitor bioapatite crystallinity changes in a number of different research fields, as archaeological, biomedical and material science.

## Methods

Sixteen fresh bone and dentine samples were obtained from different taxa: Bos taurus (cow), Sus scrofa domestica (pig), Ovis aries (sheep), Equus caballus (horse), Canis familiaris (dog), and Homo sapiens (human)^63^.

Eight archaeological bone samples come from Ateret (area E3, 1000 years CE), Megiddo (area H9, 900 years BCE), Tell es-Safi/Gath (area A3, 1200 years BCE), Neve-Yarak (area A2401, 6000 years BCE), Qesem (unclear context, late Pleistocene) archaeological sites in Israel^64^.

In Sudan the excavation of the archaeological site 16D4 (Al Khiday, Khartoum) has been carried out since 2005 within the “El Salha Archaeological Project”^65–70^. The site revealed a multi-stratified cemetery with distinct burial phases dating from the early Holocene to the beginning of the 1^st^ millennium CE^25,26,67,71–75^. Within this wide span of time, profound climatic changes occurred at regional level, from a humid environment in the early Holocene towards more arid conditions after the end of the African Humid Period^76,77^; changes of environmental and local burial conditions influenced the type and extent of diagenetic alteration of bones that were buried in different periods^25,26,75^. Because of the wide time span and the extreme conditions of diagenesis, this was considered an ideal case producing very altered and recrystallized fossil bones. A set of fourteen femurs, sampled among all burial phases, was selected as representative of the different diagenetic histories experienced during burial.

All samples were analysed by FTIR spectroscopy in transmission mode, involving KBr pelleting method for sample preparation. Each sample was lightly ground by hand using an agate mortar, then 1 mg of powdered sample was mixed with 100 mg of KBr (Sigma-Aldrich® spectroscopic grade) and pressed under 8 tons/cm^2^ pressure for 2 min in order to form a transparent pellet (12 mm in diameter). FTIR spectra were collected with a Thermo Scientific Nicolet iS 10 spectrometer; 64 scans for each spectrum were acquired in the range 4000–400 cm^−1^, with a spectral resolution of 2 cm^−1^. Spectral analysis was performed using Omnic 9 software (Thermo Scientific).

A subset of samples was analysed by X-Ray Powder Diffraction (XRPD). Analysis was performed with a PANalytical X’Pert PRO diffractometer in Bragg-Brentano geometry, equipped with a Cu X-ray tube, operating at 40 kV and 40 mA, and a X’Celerator detector. Diffractograms were acquired in the range 3–80°2θ, with a step size of 0.02°2θ and counting time of 1 s per step. Rietveld refinement^55^ analysis was performed on diffractograms using the MAUD program^78^. Instrumental contribution to line broadening was determined by measuring the NIST Si 640c standard sample, with the same experimental conditions. The crystal structure of Holly Springs hydroxyapatite^79^ was adopted as structural model for bioapatite of bone samples. A polynomial function with 6 parameters was used to describe the background, while a pseudo-Voigt function was adopted to model the diffraction profile and residual was minimized by least square method. Scale factor, background parameters, isotropic atomic displacement parameter (maintained equal for all atoms) and unit cell parameters were refined. Other atomic thermal parameters, site occupancy and atomic position remained fixed during refinement. The crystallite-size and microstrain contributions to line broadening, taking into account their anisotropy, were determined by the spherical harmonics model^80^ implemented in MAUD, by refining the spherical harmonics coefficients.

All data generated and analysed during this study are included in this article.

### Ethics statement

No animals used in this research were sacrificed for research purposes, nor are they threatened or endangered species. Fresh bones of different taxa were obtained from a slaughterhouse, dog and equine samples were obtained from a veterinary hospital and human samples were provided by Dr Sunita Ho, University of California, San Francisco, with the necessary approvals, as reported in Asscher *et al*.^63^. Fossil bones were provided by Centro Studi Sudanesi and Sub-Sahariani^26^ and by the Kimmel Centre for Archaeological Science (Weizmann Institute of Science)^64^.

## Electronic supplementary material

Supplementary Information
