# Supplementary material for: A universal curve of apatite crystallinity for the assessment of bone integrity and preservation
Source: Sci Rep. 2018 Aug 13;8:12025. doi: 10.1038/s41598-018-30642-z (PMC6089980; doi:10.1038/s41598-018-30642-z)
Supplement: Supplementary file 1 — Supplementary Information [file 41598_2018_30642_MOESM1_ESM.pdf]

A universal curve of apatite crystallinity for the assessment of bone integrity and preservation

Gregorio Dal Sasso[a], Yotam Asscher[a], Ivana Angelini[b], Luca Nodari[c], Gilberto Artioli[a]

[a]Dipartimento di Geoscienze, Università degli Studi di Padova, Via G. Gradenigo 6, 35131 Padova, Italy.

[b]Dipartimento dei Beni Culturali: archeologia, storia dell'arte, del cinema e della musica, Università degli Studi di Padova, Piazza Capitaniato 7, 35139 Padova, Italy.

[c]Istituto di Chimica della Materia Condensata e di Tecnologie per l'Energia – ICMATE – Consiglio Nazionale delle Ricerche, Corso Stati Uniti 4, 35127 Padova, Italy.

Supplementary Information

Supplementary Figure S1. FTIR spectra of analysed samples.

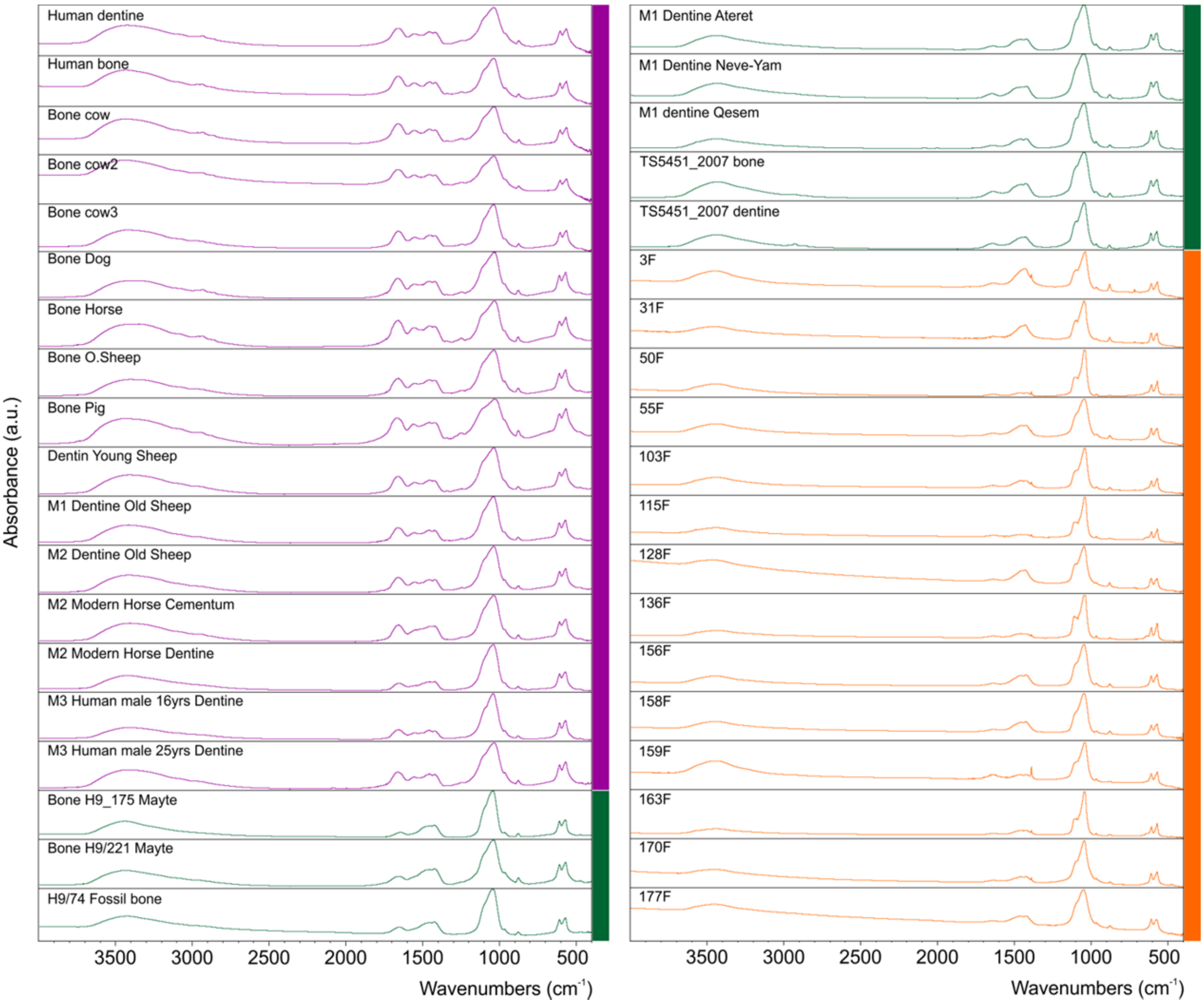

Supplementary Figure S1. The set of samples is divided in three groups: fresh bones (violet), archaeological bones from Israel (green) and archaeological bones from Sudan (orange). For each spectrum several parameters were calculated, once the baseline is established, using the method reported in the article and displayed in Fig. 1. Numerical results are reported in Supplementary Table S1, referring to the same sample code here displayed.

Supplementary Table S1. FTIR and XRPD results

Sample type	Sample code	FTIR spectroscopy					XRPD				
		FWHM (cm^{-1})	FW85% (cm^{-1})	FW80% (cm^{-1})	CO_3/PO_4	IRSF	crystallite size along c axis (nm)	microstrain along c axis	crystallite size along a axis (nm)	microstrain along a axis	Rwp (%)
Fresh bone	Human dentine	137.79	14.14	24.38	0.67	2.83	19.1	0.00002	9.7	0.00474	6.32
	Human bone	123.53	12.73	21.45	0.70	3.04	19.7	0.00016	14.3	0.00513	6.35
	Bone cow	134.64	14.42	22.24	0.69	2.89	16.1	0.00008	11.3	0.00693	4.87
	Bone cow2	135.23	13.74	22.52	0.67	2.91	17.4	0.00001	10.5	0.00750	6.02
	Bone cow3	116.83	12.57	21.12	0.69	3.01	17.2	0.00014	10.0	0.00630	6.45
	Bone Dog	133.24	12.93	20.83	0.77	3.04					
	Bone Horse	141.34	16.13	22.16	0.90	2.82					
	Bone O.Sheep	136.46	13.13	21.88	0.74	2.99					
	Bone Pig	171.42	17.58	31.95	0.83	2.85					
	Dentin Young Sheep	132.44	14.34	22.27	0.67	2.85					
	M1 Dentine Old Sheep	120.22	12.25	20.83	0.64	3.05					
	M2 Dentine Old Sheep	124.77	12.40	20.75	0.62	3.04					
	M2 Modern Horse Cementum	128.09	14.36	23.90	0.80	2.77					
	M2 Modern Horse Dentine	113.75	12.08	23.02	0.53	2.87					
	M3 Human male 16yrs Dentine	110.35	10.41	19.19	0.48	3.32					
	M3 Human male 25yrs Dentine	137.50	13.34	22.14	0.62	2.99					
Archaeological bone (Israel)	Bone H9_175 Mayte	99.22	11.00	19.42		3.13					
	Bone H9_221 Mayte	120.98	12.58	21.23		3.05					
	H9_74 Fossil bone	105.33	10.97	19.37		3.15					
	M1 Dentine Ateret	100.36	9.73	19.17		3.28					
	M1 Dentine Neve-Yam	106.55	10.90	22.27		2.95					
	M1 dENTINE Qesem	98.85	8.32	17.27		3.88					
	TS5451_2007 bone	104.01	10.66	18.13		3.37					
	TS5451_2007 dentine	89.75	8.31	14.50		4.43					
Archaeological bone (Sudan)	3F	63.95	8.45	11.51		4.71	43.7	0.00005	17.7	0.00277	5.05
	31F	57.95	7.64	14.77		5.64	60.8	0.00037	31.9	0.00202	4.92
	55F	94.69	8.59	17.42		3.74	42.5	0.00002	18.8	0.00104	5.14
	50F	47.08	7.12	8.22	0.33	6.17	61.0	0.00010	21.3	0.00265	4.45
	103F	58.74	7.74	11.36		4.78	55.0	0.00001	22.5	0.00210	4.60
	115F	44.36	6.72	8.85	0.28	6.24	67.4	0.00001	25.1	0.00232	4.83
	128F	83.20	8.61	15.96		4.60	49.5	0.00002	27.3	0.00186	4.58
	136F	56.89	6.77	8.61	0.31	6.33	71.7	0.00065	26.7	0.00218	4.43
	156F	86.68	8.55	14.65		4.00	38.6	0.00005	18.9	0.00270	5.38
	158F	83.12	8.90	14.22		3.90	34.0	0.00001	15.3	0.00332	4.73
	159F	66.17	8.27	12.61	0.48	4.66	46.9	0.00003	19.8	0.00268	4.88
	163F	43.51	6.82	8.30		6.08	65.0	0.00052	24.5	0.00237	4.77
	170F	80.92	7.91	13.67		4.68	52.4	0.00002	29.8	0.00230	5.03
	177F	92.14	8.48	19.16		3.35	44.4	0.00003	22.0	0.00195	5.01

Supplementary Table S1. Results obtained for FTIR parameters calculated in this study for the entire set of samples, i.e the full width at half maximum (FWHM) of the main phosphate peak at 1035 cm^{-1}, the width at 85% of the height of the 604 cm^{-1} peak (FW85%), the width at 80% of the height of the 565 cm^{-1} peak (FW80%), the carbonate to phosphate band intensity ratio (CO_3/PO_4) and the infrared splitting factor (IRSF). Repeated measurements provided a relative standard deviation within 5% for these parameters. XRPD analysis was carried out on a subset of samples and results obtained through Rietveld refinement are here reported: the crystallite size and microstrain along the crystallographic c axis and a axis. The agreement factor Rwp between observed and calculated diffraction pattern is also reported. The relative standard deviation associate to crystallite size determination is within 3%.

FTIR spectra of bone

FTIR spectra of bone material show major absorption bands referring to the organic matrix and to bioapatite. Those referring to the organic matrix are located in the region from 1700 to 1300 cm^{-1}, the strongest of which corresponding to the amide I (1660 cm^{-1}) and amide II (1550 cm^{-1}) vibrational bands [1].

Bioapatite shows several absorption bands relative to phosphate and carbonate groups. The phosphate strongest absorption bands corresponds to the P-O antisymmetric stretching - $\nu_3(PO_4)$ – and out-of-plane bending - $\nu_4(PO_4)$ - vibrational modes, ranging from 1200 to 900 cm^{-1} and from 700 to 500 cm^{-1}, respectively. In particular, several vibrational bands are attributed to PO_4^{3-} ions in apatitic environment (at 1035, 1045, 1056, 1061, 1075, 1090 cm^{-1}), whereas additional bands are attributed to a non-apatitic environment of phosphate and hydrogenphosphate ions (at 1020, 1100, 1114, 1151 cm^{-1}) in the $\nu_3(PO_4)$ mode [2,3]. As for the $\nu_4(PO_4)$ mode, two bands at ~604 and ~565 cm^{-1}, and a shoulder at ~575 cm^{-1}, are attributed to PO_4^{3-} ions in apatitic environment, whereas two additional bands at ~634 and ~617 cm^{-1} and one at ~550 cm^{-1} are attributed to PO_4^{3-} ions in non-apatitic environment and to HPO_4^{2-}ions, respectively [4,5]. Additionally, the apatitic OH^- libration mode can be detected at ~632 cm^{-1} [5,6]. Weaker bands corresponding to the symmetric stretching - $\nu_1(PO_4)$ - and in-plane bending -$\nu_2(PO_4)$ - vibrational modes are located at 962 cm^{-1} and 472 cm^{-1}, respectively.

Carbonate ions occur in the A and B crystallographic sites, substituting hydroxyl ions and phosphate ions, respectively, and as labile carbonate that is thought to stabilize the surface of apatite nanocrystals [7]. Carbonate absorption bands are those referring to the out-of-plane bending - $\nu_2(CO_3)$ - and antisymmetric stretching - $\nu_3(CO_3)$ - vibrational modes, detected in the range from 890 to 840 cm^{-1} and from 1600 to 1300 cm^{-1}, respectively. A more detailed distinction can be made between vibrational bands associated to carbonate ions occupying the A or B crystallographic site, i.e. carbonate ions substituting for hydroxyl (A-type substitution) or phosphate ions (B-type substitution), respectively [8–10]. Within the $\nu_3(CO_3)$ vibrational mode, bands at ~1465 and ~1542 cm^{-1} refer to the A-type carbonate and those at ~1462 and ~1415 cm^{-1} refer to the B-type carbonate; within the $\nu_2(CO_3)$ mode, bands at ~880,~872 and ~866 cm^{-1} refer to the A-type, to the B-type and labile carbonate, respectively [5,7]. It is noteworthy that vibrational bands related to the $\nu_2(CO_3)$ mode may be superimposed on a vibrational band due to the occurrence of HPO_4^{2-}ions in bioapatite [7].

Spectral analysis and selection of suitable parameters from FTIR spectra

A subset of samples (13), covering the entire range of crystallinity degree and preservation state, was selected for spectral analysis using the deconvolution method. Since our main interest is focused on the $\nu_4(PO_4)$ vibrational mode, the spectrum region from 900 to 400 cm^{-1} was selected. In Supplementary Figure S2a two extreme cases are reported as an example, showing the spectra (and their deconvolution) of a fresh bone and a heavily altered archaeological bone. Results were used to evaluate the effectiveness of the parameters calculated directly from the spectra. The physical meaning of the vibrational bands selected for the deconvolution process is already reported in previously published papers [4]; the selected bands are those at 604, 565 and 575 cm^{-1}, attributed to PO_4^{3-} ions in apatitic environment, and those at 634, 617 and 550 cm^{-1} attributed to PO_4^{3-} ions in non-apatitic environment and to HPO_4^{2-} and OH^- ions. All bands were modelled with a Gaussian curve. The same linear baseline used to calculate the IRSF and FW85% parameters (as described in the "FTIR spectral analysis" section of the article) is here selected. Nonlinear least square data fitting is performed using the algorithm implemented in MagicPlot pro 2.7.2 and the fit function results from the sum of the fit curves. The quality of the fit was monitored by the coefficient of determination R^2 (Supplementary Table S2). The width (reported ad half width at half maximum – HWHM) and the amplitude of the deconvoluted 604 cm^{-1} peak were compared to the FW85% parameter (the non-deconvoluted width at 85% of the height) and to the intensity of the 604 cm^{-1} peak (as measured for the IRSF calculation, corresponding to **a** in Fig. 1), respectively (Supplementary Fig. S2). In both cases an extremely good linear relationship can be observed between the deconvoluted and non-deconvoluted peaks width and between the deconvoluted and non-deconvoluted peaks amplitude, described through linear regression models with a high coefficient of determination R^2 (0.99 and 0.98, respectively). Even though absolute values of width and amplitude differs, depending on the method used to calculate them, both the deconvolution and non-deconvolution approaches provide consistent results, showing that the proposed parametrization carries physically significant information and enables a reliable inter-sample comparison.

Supplementary Figure S2. Comparison between parameters obtained from deconvoluted and non-deconvoluted spectra.

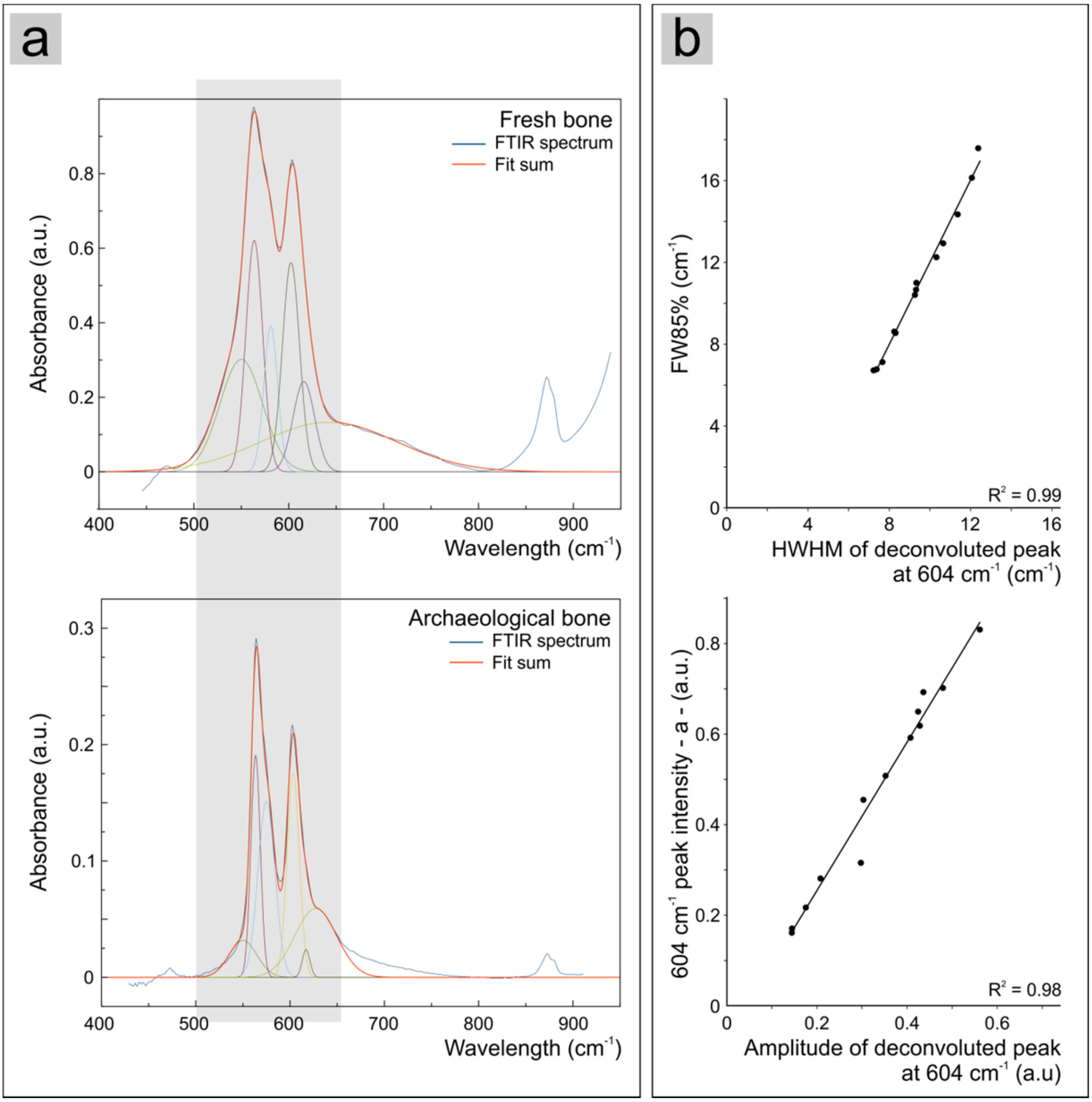

Supplementary Figure S2. a. Example of FTIR spectra of a fresh bone (top) and an archaeological bone (bottom), showing the deconvoluted vibrational bands and the resulting curve fit; the fit interval is highlighted in light grey; **b.** Linear correlations between: (top) the FW85% parameter, measured without peaks deconvolution, and the width (half width at half maximum) of the deconvoluted peak at 604 cm^{-1}; (bottom) the non-deconvoluted 604 cm^{-1} peak intensity measured from the baseline and the amplitude of the deconvoluted peak at 604 cm^{-1}.

Supplementary Table S2. Comparison between parameters obtained from deconvoluted and non-deconvoluted spectra.

Sample type	Sample code	FW85% (cm^{-1})	604 cm^{-1} peak intensity (a.u.)	Deconvoluted 604 cm^{-1} peak		R^2
				HWHM (cm^{-1})	amplitude (a.u.)	
Fresh bone	Bone Dog	12.93	0.83	10.65	0.56	0.9996
	Bone Horse	16.13	0.59	12.07	0.41	0.9991
	Bone Pig	17.58	0.69	12.38	0.44	0.9996
	Dentin Young Sheep	14.34	0.70	11.37	0.48	0.9996
	M1 Dentine Old Sheep	12.25	0.51	10.33	0.35	0.9760
	M3 Human male 16yrs Dentine	10.41	0.65	9.27	0.42	0.9996
Archaeological bone (Israel)	Bone H9_175 Mayte	11.00	0.45	9.34	0.30	0.9996
	TS5451_2007 bone	10.66	0.62	9.32	0.43	0.9995
Archaeological bone (Sudan)	50F	7.12	0.22	7.67	0.18	0.9963
	115f	6.72	0.16	7.23	0.14	0.9684
	128f	8.61	0.17	8.25	0.15	0.9991
	136f	6.77	0.32	7.38	0.30	0.9823
	156f	8.55	0.28	8.32	0.21	0.9993

Supplementary Table S2. The FW85% parameter and the 604 cm^{-1} peak intensity (referring to **a** when calculating the IRFS parameter – Fig. 1) are associated to the width (half width at half maximum –HWHM) and to the amplitude of the deconvoluted peak at 604 cm^{-1} for a subset of samples covering the entire range of crystallinity degree. The coefficient of determination R^2 resulting from the deconvolution process is also reported.

References

1. Lebon, M. *et al.* New parameters for the characterization of diagenetic alterations and heat-induced changes of fossil bone mineral using Fourier transform infrared spectrometry. *J. Archaeol. Sci.* **37,** 2265–2276 (2010).
2. Lebon, M., Reiche, I., Fröhlich, F., Bahain, J. J. & Falguères, C. Characterization of archaeological burnt bones: Contribution of a new analytical protocol based on derivative FTIR spectroscopy and curve fitting of the ν 1 ν 3 PO4 domain. *Anal. Bioanal. Chem.* **392,** 1479–1488 (2008).
3. Rey, C., Shimizu, M., Collins, B. & Glimcher, M. J. Resolution-enhanced fourier transform infrared spectroscopy study of the environment of phosphate ion in the early deposits of a solid phase of calcium phosphate in bone and enamel and their evolution with age: 2. Investigations in thev 3 PO4 domain. *Calcif. Tissue Int.* **49,** 383–388 (1991).
4. Rey, C., Combes, C., Drouet, C., Sfihi, H. & Barroug, A. Physico-chemical properties of nanocrystalline apatites: Implications for biominerals and biomaterials. *Mater. Sci. Eng. C* **27,** 198–205 (2007).
5. Rey, C., Combes, C., Drouet, C. & Grossin, D. *Bioactive Ceramics: Physical Chemistry. Comprehensive Biomaterials* (Elsevier Ltd., 2011). doi:10.1016/B978-0-08-055294-1.00178-1
6. Miller, L. M. *et al.* In situ analysis of mineral content and crystallinity in bone using infrared micro-spectroscopy of the nu(4) PO(4)(3-) vibration. *Biochim. Biophys. Acta* **1527,** 11–19 (2001).
7. Rey, C., Collins, B., Goehl, T., Dickson, I. R. & Glimcher, M. J. The carbonate environment in bone mineral: A resolution-enhanced fourier transform infrared spectroscopy study. *Calcif. Tissue Int.* **45,** 157–164 (1989).
8. LeGeros, R. Z. Apatites in biological systems. *Prog. Cryst. Growth Charact.* **4,** 1–45 (1981).
9. Sponheimer, M. & Lee-Thorp, J. a. Alteration of enamel carbonate environments during fossilization. *J. Archaeol. Sci.* **26,** 143–150 (1999).
10. Wopenka, B. & Pasteris, J. D. A mineralogical perspective on the apatite in bone. *Mater. Sci. Eng. C* **25,** 131–143 (2005).
